# Internet Use as a Moderator of the Relationship Between Personal Resources and Stress in Older Adults: Cross-Sectional Study

**DOI:** 10.2196/52555

**Published:** 2024-07-19

**Authors:** Angélique Roquet, Paolo Martinelli, Charikleia Lampraki, Daniela S Jopp

**Affiliations:** 1 Institute of Psychology University of Lausanne Lausanne Switzerland; 2 Department of Psychology, University of Geneva Geneva Switzerland

**Keywords:** internet use, aging, stress, personal resources, technical resources, Hobfoll’s Conservation of Resources theory, COR theory

## Abstract

**Background:**

Internet use has dramatically increased worldwide, with over two-thirds of the world’s population using it, including the older adult population. Technical resources such as internet use have been shown to influence psychological processes such as stress positively. Following the Conservation of Resources theory by Hobfoll, stress experience largely depends on individuals’ personal resources and the changes in these resources. While personal resource loss has been shown to lead to stress, we know little regarding the role that technical resources may play on the relationship between personal resources and stress.

**Objective:**

This study aims to investigate the moderating effect of technical resources (internet use) on the relationship between personal resources and stress in younger and older adults.

**Methods:**

A total of 275 younger adults (aged 18 to 30 years) and 224 older adults (aged ≥65 years) indicated their levels of stress; change in personal resources (ie, cognitive, social, and self-efficacy resource loss and gain); and internet use. Variance analyses, multiple regression, and moderation analyses were performed to investigate the correlates of stress.

**Results:**

Results showed that older adults, despite experiencing higher levels of resource loss (questionnaire scores: 1.82 vs 1.54; *P*<.001) and less resource gain (questionnaire scores: 1.82 vs 2.31; *P*<.001), were less stressed than younger adults (questionnaire scores: 1.99 vs 2.47; *P*<.001). We observed that the relationship among resource loss, resource gain, and stress in older adults was moderated by their level of internet use (β=.09; *P*=.05). Specifically, older adults who used the internet more frequently were less stressed when they experienced high levels of both loss and gain compared to their counterparts who used internet the less in the same conditions. Furthermore, older adults with low resource gain and high resource loss expressed less stress when they used the internet more often compared to those with low internet use.

**Conclusions:**

These findings highlight the importance of internet use in mitigating stress among older adults experiencing resource loss and gain, emphasizing the potential of digital interventions to promote mental health in this population.

## Introduction

### Background

Internet use has increased drastically in the last decade, with two-thirds of the world’s population now being familiar with it [1]. For example, in Switzerland, 90% of people aged >15 years use the internet daily, and even 70.3% of people aged >65 years use it regularly [2]. However, few studies have explored the role of internet use on psychological processes such as stress in the context of personal resource loss and its age-related specificities.

Older adults are often confronted with a variety of challenges that can result in social, health, and cognitive losses. Specifically, older adults may experience reduced social support networks, decreased physical functioning, and a decline in cognitive abilities such as memory and executive functioning [3,4]. These losses can have a negative impact on overall well-being, including increased levels of stress, depression, and anxiety [5,6]. Moreover, individuals tend to place greater value on avoiding losses than on acquiring gains and, as a result, are often more motivated to take action to avoid potential losses than to pursue potential gains. Technical resources can serve as an additional resource that helps older adults maintain or improve their level of mental health in various ways [7,8], such as through web-based social interaction, web-based counseling, health services, or cognitive stimulation. In other words, technical resources can help older adults reach their maintenance and loss managements goals.

In this study, we investigated the effect of technical resources, specifically internet use, and the extent to which they can buffer the relationship between personal resources and stress in both younger and older adults.

### Age-Related Differences in Personal Resources and Their Impact on Stress Levels

Personal resources are typically considered attributes that individuals value and that enhance their ability to function effectively in terms of controlling and impacting their environment [9,10]. Moreover, individuals’ personal resources, including their health, social support, and financial means, along with their mental strengths such as self-efficacy, change over the course of life due to a combination of factors, including biological aging, life experiences, and environmental factors. According to the life span theory, personal resources tend to decrease as individuals age, putting older individuals at risk of decline in overall well-being [11]. For instance, age-related changes such as declining social networks, poor mobility, retirement, and development of chronic illness can contribute to social isolation and feelings of loneliness in older adults [12-14], which have been associated with increased stress and poor well-being [15-17].

Various theories suggest that older adults may develop compensatory strategies to manage the decline in personal resources and maintain well-being [5,18-22]. According to the Conservation of Resources (COR) theory by Hobfoll [20], individuals seek to gain new resources to maintain or enhance their well-being, particularly in the face of stress and adversity [6]. More specifically, when individuals experience a loss of resources, such as a decline in health or social support, they may be more vulnerable to stress and negative well-being outcomes. However, if they can gain new resources, this can help offset the negative impact of the loss and buffer against the effects of stress. Thus, while resource losses can have a significant negative impact on individuals’ well-being, resource gains can help replenish those losses and promote resilience.

For older adults, resource gains may involve engaging in activities such as taking classes, volunteering, participating in social activities, or learning new skills. Specifically, technical resources provide a support to access new activities [23]. However, the extent to which internet use influences the relationship between personal resources and stress in aging is not yet fully understood. This study aimed to better understand how the internet can be used as a resource to support the well-being of older adults, particularly in the context of stress and aging.

### Internet Use Role as a Resource Gain in the Context of Older Adults’ Stress

Internet use can facilitate the gain of resources, providing older adults with additional means to cope with stress and improve their mental health outcomes [24-29]. For instance, the internet can serve as a platform for social support, information seeking, and engaging in meaningful activities, all of which can contribute to better mental health outcomes in later life [23]. More specifically, higher levels of internet use predict higher levels of social support, reduced loneliness, and better life satisfaction and psychological well-being among older adults [26].

For example, by using the internet for social interactions, older adults can increase their social networks, receive emotional support, and build relationships with others [28-30] to reduce social isolation and stress. For example, Li et al [31] examined the relationship among social isolation, cognitive functioning, depression, and internet use among older adults. The results showed that social isolation was significantly associated with poorer cognitive functioning and higher levels of depression among older adults. Moreover, internet use moderated the relationship between social isolation and cognitive functioning, suggesting that internet use may have a protective effect on cognitive functioning among socially isolated older adults. Finally, the results showed that internet use was associated with lower levels of depression among older adults regardless of their level of social isolation. This suggests that internet use could serve as a protective factor for cognitive functioning and that it represents an important factor for improving mental health outcomes among older adults.

Moreover, through information research using the internet, older adults can gain knowledge and skills to manage stressors that arise in later life. Being confronted with or anticipating age-related loss of physiological functioning, older adults are interested in acquiring health knowledge [32-34]. Higher web-based health literacy is associated with more positive health behaviors and better health knowledge and attitudes in older adults [35]. In addition, engaging in other meaningful activities such as web-based learning, gaming, shopping, and hobbies can also have positive effects on the psychological outcomes of older adults [36-41]. For example, Gallistl and Nimrod [36] examined the relationship between internet use for leisure activities and well-being among older adults. The results showed that older adults who used the internet for leisure activities reported higher levels of subjective well-being, social connectedness, and life satisfaction than those who did not use the internet for these activities. Moreover, several studies have reported that web-based gaming improves older adults’ physical and cognitive functioning [42,43], social interaction, and enjoyment and decreases social isolation [44-46]. These studies commonly suggest that, by participating in web-based activities, older adults can gain new skills, challenge themselves, increase their social networks, and find enjoyment in their free time.

Little research has been conducted on the relationship among personal resources, internet use, and stress at different ages. However, determining the potential benefits of internet use in managing stress in later life may highlight the importance of promoting access to and use of technology among older adults.

### This Study

The main objective of this study was to better understand the underlying mechanisms contributing to age-related differences in stress as a function of changes in personal resources and internet use. More specifically, we investigated (1) age-related differences in stress and its associated predictors; (2) whether the level of resource gains buffered the relationship between resource losses and stress in younger and older adults, replicating the findings by Hobfoll [20]; and (3) whether the profile of internet use in younger and older adults moderated the relationship between resource gains and losses and stress levels.

First, we tested the hypothesis that stress levels differed by age group. We expected that older adults would report lower levels of stress compared to younger adults. This hypothesis was based on previous research that has consistently demonstrated that older adults are exposed to fewer stressors than younger adults [46,47], leading to better well-being outcomes such as less stress [48-50].

The second set of hypotheses concerned the replication of the COR theory [20], describing that resource losses would have a considerably stronger impact than resource gains on individuals’ stress perception [6]. Moreover, COR theory explains that resource gains buffer the effect of resource losses on stress [20]. Accordingly, we expected that (1) more resource losses would be associated with a higher level of stress; (2) resources losses would have a stronger impact on stress than resource gains; and (3) the relationship between resource losses and stress would be moderated by the level of resource gains, with higher levels of gains helping buffer the negative impact of losses on stress levels.

Finally, we hypothesized that the moderating effect of resource gains on the relationship between resource losses and stress would vary based on levels of internet use, presenting distinct profiles for the younger and older adults. First, given previous findings showing that internet use may have a positive impact on older adults’ well-being and stress levels [51-53], we expected that internet use would moderate the relationship between resource gains and losses and stress—individuals who reported higher levels of internet use would experience a greater protective effect of resource gains (ie, stronger effect of gains) than those who reported lower levels of internet use. This hypothesis was based on previous studies demonstrating that higher use of the internet was associated with higher levels of stress, depression, loneliness, and anxiety in young adults [54,55].

## Methods

### Procedures and Participants

We conducted a cross-sectional study in the French-speaking part of Switzerland. The participants were native or fluent French speakers. We recruited 510 individuals, of whom 280 (54.9%) were aged <30 years (mean age 25.00, SD 2.09 y) and 230 (45.1%) were aged >65 years (mean age 73.55, SD 7.16 y; see Table 1 for detailed participant characteristics). Younger participants were mainly undergraduates from the University of Lausanne, whereas the rest of the participants were recruited using the snowball sampling technique [56]. Recruited individuals volunteered to participate in the study and were not remunerated. For being included, participants had to be able to speak and understand French and had to be aged between 18 and 30 years or >65 years. Participants filled out a web-based open questionnaire containing questions on stress and potential predictors or moderators such as personal resources and internet use (ie, questions presented in a specific order).

The sample size used in this experiment was based on an a priori power analysis conducted in G*Power (version 3.1) [57]. We assumed an effect size of Cohen *f*=0.06, which was derived from previous relevant studies on the buffer effect of resource gains on the association between resource losses and stress [58,59], and an α of .05. Specifically, a total sample size of 404 participants (n=202 per group) provided 90% power to detect effects. To exceed this criterion and achieve >80% power, we recruited 510 participants (ie, n=280, 54.9% younger adults and n=230, 45.1% older adults).

**Table 1:** Participant characteristics (N=510).

Variable			Younger adults (n=280)		Older adults (n=230)		Chi-square test for group comparison		
							Chi-square (*df*)		*P* value
Age (y), mean (SD; range)			25.00 (2.09; 18-29)		73.55 (7.16; 65-98)		—^a^		—
**Gender, n (%)**									
	Women	190 (67.9)		130 (56.5)		6.9 (1, 510)		.01	
	Men	90 (32.1)		96 (41.7)		5.0 (1, 510)		.03	
**Educational level^b^, n (%)**									
	Obligatory school not finished	1 (0.4)		16 (7)		17.1 (1, 510)		<.001	
	Obligatory school	4 (1.4)		51 (22.2)		56.5 (1, 510)		<.001	
	Professional formation	18 (6.4)		79 (34.3)		63.9 (1, 510)		<.001	
	General education	4 (1.4)		5 (2.2)		0.4 (1, 510)		.56	
	Professional maturity	15 (5.4)		10 (4.3)		0.3 (1, 510)		.60	
	Gymnasium maturity	49 (17.5)		9 (3.9)		24.0 (1, 510)		<.001	
	Specialized university	15 (5.4)		20 (8.7)		2.2 (1, 510)		.14	
	University	173 (61.8)		37 (16.1)		108.9 (1, 510)		<.001	
	Doctoral degree	0 (0)		3 (1.3)		3.7 (1, 510)		.06	
**Financial adequacy, n (%)**									
	More money than needed	54 (19.3)		59 (25.7)		3.2 (1, 510)		.08	
	Enough money	177 (63.2)		158 (68.7)		1.3 (1, 510)		.25	
	Less money than needed	48 (17.1)		13 (5.7)		15.6 (1, 510)		<.001	

^a^Chi-square analyses were not conducted on participants’ mean ages.

^b^The term *obligatory school not finished* corresponds to <11 years of education; *obligatory school* corresponds to 11 years of education; *professional formation, general education, professional maturity, and gymnasium maturity* correspond to 4 additional years of education; *university and specialized university* correspond to 3 to 5 additional years of education; and *doctoral degree* corresponds to 3 to 5 additional years of education.

### Ethical Considerations

This study was approved by the Social and Political Sciences Ethics Committee of the University of Lausanne (C-SSP-092022-00002). Written informed consent was obtained from participants before the questionnaire was made available, and they were informed that they could decide to quit the study at any point. This ensured that participants were well informed of the study’s objectives and the potential impact of their contribution. Furthermore, participants were made aware of the duration of their involvement, which entailed completing a web-based questionnaire lasting approximately 30 minutes (ie, the questionnaire comprised 27 pages with 15 items per page and the possibility to go back).

Before deployment, the questionnaire was tested to ensure its feasibility, enhancing its clarity and ease of completion. For nonapplicable items or when participants chose not to respond, options such as “not applicable” or “prefer not to say” were provided, respecting participant autonomy while preserving data integrity. In addition, to ensure valid responses, at least one answer selection per question was mandated, minimizing incomplete or inconsistent submissions and maintaining data reliability.

The informed consent process outlined the data management protocols, including the types of data collected, the methodologies used for data treatment using SPSS (IBM Corp), and the storage solutions provided by Switch Drive (Switch). A commitment was made to the participants that their data would be anonymized and held confidentially, with plans for eventual sharing in an open-access data repository (eg, SWISSUbase for 5 years) after the removal of any personally identifiable information. This study was developed using SurveyMonkey (for the Checklist for Reporting Results of Internet E-Surveys, see Multimedia Appendix 1), a web-based survey platform known for its ease of use and robust data analysis tools. It allows for the creation, distribution, and analysis of surveys, making it an ideal choice for collecting detailed feedback and insights. In addition, SurveyMonkey’s strong emphasis on data security and privacy ensures the integrity and confidentiality of the data collected in the study. Multiple submissions were controlled by monitoring IP addresses and the anonymous codes assigned to each participant in addition to checking for consistency in the responses. Moreover, analyses were performed on questionnaires that were fully completed. No monetary compensation was provided to participants upon the completion of the questionnaire.

### Measures

#### Predictors

##### Sociodemographic Variables

Demographic variables included age (in years), gender (0=*men*; 1=*women*), educational level (1=*obligatory school not finished*, 2=*obligatory school*, 3=*professional formation*, 4=*general education*, 5=*professional maturity*, 6=*gymnasium maturity*, 7=*specialized university*, 8=*university*, and 9=*doctoral degree*), and financial adequacy (1=*more money than needed*, 2=*enough money*, and 3=*less money than needed*).

##### Personal Resources

Personal resources were assessed using the 13-item Personal Resource Questionnaire–Short Form [60]. The short version of the questionnaire includes items concerning cognition, self-efficacy, and social relations. For this study, we used 2 parts of the questionnaire: losses (“To what extent did the listed resources decrease in the last year?”) and gains (“To what extent did the listed resources increase in the last year?”; a total of 13 items × 2 = 26 items; for details, see Table 2). Each item was evaluated on a 5-point Likert scale ranging from 1=*none* to 5=*great amount*. Mean composite scores were calculated for losses and gains. Specifically, we created 2 types of indicators (ie, domain-general resources=resource gains and losses) and 3 types of resources in each condition (ie, domain-specific resources=self-efficacy, cognition, and social resources). Lower scores indicate lower levels of each personal resource condition.

**Table 2:** Details of the Personal Resource Questionnaire used in this study.

Resource	Number of items	Items	Cronbach α	
			Gains	Losses
Cognition	5	“Sound cognitive functioning” “Intelligence” “Good memory ability” “Ability to concentrate” “Ability to think and understand quickly”	0.96	0.93
Self-efficacy	4	“Sense of control over my life” “Ability to control my future” “Ability to achieve my goals” “Ability to put my plans into action”	0.95	0.92
Social relations	4	“Companionship of other people” “Close relationship to at least one friend” “Positive relationship partner” “Close relationship to one or more family members”	0.79	0.64

##### Internet Use

Internet use was assessed using the 8-item Mobile Device Proficiency Questionnaire [61] measuring participants’ ability to perform on the internet using a mobile device (example item: “Using a mobile device I can read the news on the Internet?”). Each item was scored on a 5-point Likert scale ranging from 1=*never tried* to 5=*very easily*. A mean composite score was calculated, with lower scores indicating low levels of internet use. The Cronbach α for this study was 0.96.

#### Outcome Variable: Stress

Participants’ stress level was assessed using the 5-item Perceived Stress Scale by Cohen et al [62] (eg, “In the last month, how often have you felt anxious and stressed?”). Each item was scored on a 4-point Likert scale ranging from 0=*never* to 4=*very often*. We calculated a mean composite score in which lower values indicated a less frequent experience of stress in the last month. The Cronbach α was 0.80.

### Analytical Strategy

Differences between younger and older adults were first tested on stress and its predictors or moderators (eg, personal resources) using between-group ANOVAs. We then conducted correlation analyses to gain a better understanding of relationships among age, gender, educational level, financial adequacy, personal resources, internet use, and stress and prepare a more complex moderation analysis.

Second, to replicate the findings by Hobfoll and Lilly [6] and Hobfoll [21] regarding the stronger effect of resource losses than resource gains on stress, we conducted simple regression using participants’ characteristics and personal resource variables as predictors. Moreover, to explore the moderating effect of resource gains on the relationship between resource losses and stress levels, we conducted moderation analyses using PROCESS (version 3.5) by Hayes [63], model 1. These analyses allowed for the examination of how the relationship between resource losses and stress levels varied depending on the level of resource gains reported by participants (ie, effect of the 2-way interaction gains × losses on stress).

Finally, to determine whether internet use influenced the moderation effect of resource gains on the association between resource losses and stress, a moderation analysis was performed using model 3 on PROCESS (ie, version 3.5 for SPSS by Hayes [63]). This method allowed for the testing of the effect of the triple interaction, internet use × gains × losses, on stress. In all moderation analyses, we controlled for age, gender, educational level, and financial adequacy.

In each regression and moderation analysis, we divided the analysis into 2 sets: the first set included the domain-general resources as general gains and losses, and the second set included the gains and losses of domain-specific resources such as social, cognition, and self-efficacy.

Unstandardized coefficients and 95% CIs were reported. The level of statistical significance was set at *P*<.05. All analyses were performed using SPSS (version 26; IBM Corp).

## Results

### Mean-Level Comparisons

Mean-level tests were conducted to determine the age-related differences in stress and personal resources (Table 3).

The results showed that older adults were less stressed than younger adults (1.99 vs 2.47; *P*<.001; ie, questionnaire scores). Older adults had more losses compared to younger adults (1.82 vs 1.54; *P*<.001; ie, questionnaire scores), whereas they presented less resource gains (1.82 vs 2.31; *P*<.001; ie, questionnaire scores). More specifically, the analyses conducted on domain-specific resources (ie, social, self-efficacy, and cognition) revealed that the resource losses in the social, self-efficacy, and cognitive domains tended to increase with age, whereas the social, self-efficacy, and cognitive resource gains tended to decrease with age. Finally, older adults reported less internet use than younger adults (3.35 vs 4.91; *P*<.001; ie, questionnaire scores).

**Table 3:** Younger and older adults’ mean stress and personal resource variables (ie, questionnaire scores).

					Younger adults (n=275), mean (SD)			Older adults (n=224), mean (SD)			Test for mean-level differences (N=499)			
											*F* test (*df*)			*P* value
Stress					2.47 (0.70)			1.99 (0.69)			58.45 (1, 497)			<.001
**Resources**														
	**Losses**			1.54 (0.63)			1.82 (0.76)			14.97 (1, 497)			<.001	
		Social	1.54 (0.63)			1.60 (0.72)			1.07 (1, 497)			.30		
		Self-efficacy	1.74 (0.92)			1.90 (0.93)			3.62 (1, 497)			<.06		
		Cognition	1.47 (0.73)			1.92 (0.88)			39.27 (1, 497)			<.001		
	**Gains**			2.31 (0.97)			1.82 (0.95)			32.72 (1, 497)			<.001	
		Social	2.57 (1.04)			2.00 (1.02)			37.37 (1, 497)			<.001		
		Self-efficacy	2.41 (1.14)			1.82 (1.06)			34.79 (1, 497)			<.001		
		Cognition	2.03 (1.08)			1.67 (0.99)			15.57 (1, 497)			<.001		
Internet use					4.91 (0.23)			3.35 (1.38)			342.74 (1, 497)			<.001

### Correlation Analysis

We performed correlational analyses to highlight relationships among sociodemographics, independent variables (domain-general and domain-specific resources and internet use), and stress (Table 4; for a complete table of correlations, see Multimedia Appendix 2).

In the total sample group, age, gender, and financial adequacy were correlated with stress; being female, having less money than needed, and being younger were associated with higher stress. Moreover, resource losses were related to higher stress. Specifically, higher stress was associated with more social, cognition, and self-efficacy losses. Participants who used the internet more often reported higher levels of stress.

In the separate age group analyses, we found that higher levels of stress were associated with higher levels of resources losses and, more specifically, social, cognitive, and self-efficacy losses in both younger and older adults. Moreover, correlations in the younger adult group revealed that being female and having less money than needed were associated with higher stress. Furthermore, higher levels of domain-general resource gains in younger adults were associated with stress, and domain-specific resource gains such as cognitive and self-efficacy were related to being less stressed. Concerning the older adult group, analyses revealed that having a higher level of education was associated with lower stress. Finally, while the use of the internet was not associated with stress scores among the younger participants, it was negatively associated with stress among the older participants, indicating that more internet use was linked to lower stress levels.

**Table 4:** Significant Pearson correlations (r) between participant characteristics and personal resources and stress levels for the total sample (younger and older adults; N=510).

				Younger adults (n=280)				Older adults (n=230)					Total sample		
				*r*		*P* value		*r*		*P* value		*r*			*P* value
Age				–0.01		.81		0.02		.81		–0.32			<.001
Gender				0.21		<.001		0.11		.08		0.20			<.001
Educational level				–0.03		.59		–0.25		<.001		0.08			.08
**Financial adequacy**				0.28		<.001		0.12		.08		0.25			<.001
	**Losses**			0.42		<.001		0.36		<.001		0.30			<.001
		Social	0.21		<.001		0.21		.002		0.18			<.001	
		Self-efficacy	0.49		<.001		0.40		<.001		0.40			<.001	
		Cognition	0.30		<.001		0.33		<.001		0.20			<.001	
	**Gains**			–0.21		<.001		–0.01		.87		–0.03			.49
		Social	–0.08		.18		–0.01		.81		0.04			.41	
		Self-efficacy	–0.28		<.001		–0.01		.85		–0.07			.12	
		Cognition	–0.18		.003		–0.00		.97		–0.04			.35	
Internet use				–0.05		.39		–0.17		.01		0.12			.005

### Moderation Analyses

#### Overview

Regarding the profiles of internet use between younger and older adults, we found notable differences. Specifically, younger adults exhibited uniformly high use rates (median 5.00, range 3.38-5.00), whereas older adults demonstrated a considerable range in their internet use behavior, from very low to very high (median 3.56, range 1.00-5.00). To evaluate the moderating effect of age on the link between resource loss, resource gain, and internet use on stress, we initially tested a model including the age group variable in a quadruple interaction term (loss × gain × internet use × age groups; not presented). However, no significant interaction was found (β=–.02; *P*=.95), possibly due to the complexity of the interaction term and the unequal variances in internet use across age groups. Indeed, the assumption of homogeneity of variances for internet use was not respected (Levene test: *F*_1,502_=536.63; *P*<.001), showing a difference in variances across groups. On the basis of these findings but also on previous research in the field suggesting that internet use may influence the level of stress, we decided to present the following analysis separately for younger and older individuals (although the results should be interpreted with caution). This methodological approach was crucial for understanding how internet use influenced the relationship between resource loss and gain and stress, allowing for an exploration of use trajectories characteristic of younger and older adults without the confounding influence of the homogeneous high internet use found in the younger cohort.

#### Domain-General Resources

First, simple regression (model 1; Multimedia Appendix 3) revealed that resource indicators accounted for 32% and 20% of the individual differences in stress levels in younger and older adults, respectively. Similarly, for younger and older adults, the strongest predictor was resource losses (younger adults: β=.45 and *P*<.001; older adults: β=.37 and *P*<.001) followed by resource gains (younger adults: β=–.20 and *P*<.001; older adults: β=–.13 and *P*=.01), suggesting that individuals who experienced higher resource losses and lower gains tended to feel more stressed as compared to those with lower levels of losses and higher levels of gains.

**Figure 1:**
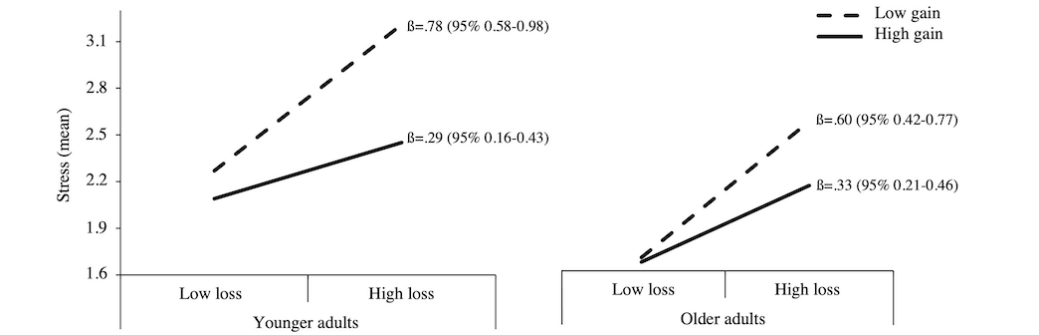
Mean stress depending on the level of gains (high vs low) and losses (high vs low) in younger and older adults. The slopes’ values represent the coefficients and 95% CIs.

Second, we found a significant 2-way interaction (model 2; Multimedia Appendix 3 and Figure 1) between resource gains and resource losses on levels of stress as the dependent variable in both younger and older adults, confirming our second hypothesis (β=.26 and β=.16 for younger and older adults, respectively). Specifically, individuals with high levels of resource gains who also reported high levels of losses felt less stressed compared to individuals with lower levels of resource gains and higher levels of resource losses.

Finally, internet use influenced the moderation effect of resource gains on the relationship between resource losses and stress, as seen in a significant 3-way interaction among internet use, gains, and losses in older adults (model 3; Multimedia Appendix 3; β=.09). More specifically, compared to the second model, the third model presented an increase in the index of adjustment (ie, a change in explained variance) of 0.05 (Δ*R*^2^=0.05; *P*=.002). The 3-way interaction was not significant in younger adults (β=.22).

**Figure 2:**
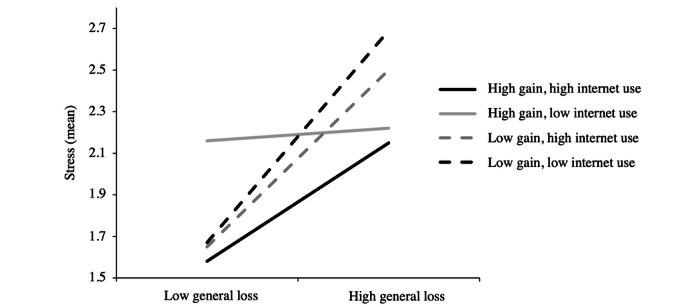
Mean stress depending on the level of gains (high vs low), internet use (high vs low), and losses (high vs low) in older adults.

To further the understanding of the 3-way interaction in older adults, we examined the conditional effects of resource losses at 1 SD above (+1 SD) and 1 SD below (–1 SD) the mean scores of resource gains (first moderator) and internet use (second moderator; Table 5 and Figure 2). As the 3-way interaction (losses × gains × internet use) was not significant in younger adults, we reported only the conditional effects of older adults. The results showed significant moderation effects of resource gains and internet use on the relationship between domain-general losses and stress. Specifically, significant moderation effects were observed only in cases in which individuals reported high levels of gains and high levels of internet use (β=.37), as well as in cases in which individuals reported low levels of gains and high levels of internet use (β=.55) and low levels of both gains and internet use (β=.66). These results suggest that the use of the internet reinforces the buffering effect of gains on the relationship between resource losses and stress.

According to the third hypothesis, we conducted an additional analysis to examine the differences between individuals with high internet use and those with low internet use in relation to their gains and losses (Table 6). As the 3-way interaction (losses × gains × internet use) was not significant in younger adults, we reported only the conditional effects of older adults. The results showed that internet use effects (ie, differences between participants with a higher internet use and participants with a lower internet use) were marginally significant only in cases in which individuals reported high levels of gains and low levels of losses (β=–.21).

**Table 5:** Conditional effects of domain-general resource losses (ie, independent variable) at +1 SD and –1 SD of the mean scores of gains and internet use (ie, moderators) in older adults.

	β coefficient (SE; 95% CI)	*P* value
Losses at –1 SD of gains and –1 SD of internet use	0.66 (0.12; 0.34 to 0.89)	.001
Losses at –1 SD of gains and +1 SD of internet use	0.55 (0.14; 0.28 to 0.83)	.001
Losses at +1 SD of gains and –1 SD of internet use	0.04 (0.17; –0.30 to 0.37)	.84
Losses at +1 SD of gains and +1 SD of internet use	0.37 (0.08; 0.20 to 0.53)	.001

**Table 6:** Conditional effects of internet use (ie, independent variable) on stress at +1 SD and –1 SD of the mean scores of resource losses and gains (ie, moderators) in older adults.

	β coefficient (SE; 95% CI)	*P* value
Internet use at –1 SD of gains and –1 SD of losses	–0.01 (0.06; –0.12 to 0.10)	.89
Internet use at –1 SD of gains and +1 SD of losses	–0.07 (0.08; –0.23 to 0.09)	.40
Internet use at +1 SD of gains and –1 SD of losses	–0.21 (0.12; –0.44 to 0.02)	.08
Internet use at +1 SD of gains and +1 SD of losses	–0.03 (0.06; –0.14 to 0.09)	.67

#### Domain-Specific Resources

Similarly to the domain-general resources, we conducted 3 regression models for the domain-specific resources in both younger and older adults (self-efficacy, cognition, and social resources; Multimedia Appendix 4). The first model showed that resource losses were the strongest predictor of stress levels across different age groups regardless of the specific type of resource considered. The coefficient of losses for self-efficacy was found to be the highest in both younger and older adults, with more losses being related to higher levels of stress in both groups. Moreover, higher gains in self-efficacy, cognition, and social resources significantly predicted lower stress levels in younger adults. However, in older adults, the only significant predictor was self-efficacy gains, with more gains being associated with less stress. These findings suggest that different types of resource gains may play varying roles in shaping stress experiences across different age groups.

Moreover, the impact of resource losses on stress in younger and older adults was significantly influenced by their levels of gains, which varied depending on the type of resource. Those with high levels of self-efficacy and cognition gains tended to experience less stress when they also presented high levels of self-efficacy and cognition losses (younger adults: unstandardized coefficient β_Cognition_=.21, 95% CI 008-0.34 and β_Self-efficacy_=.20, 95% CI 0.08-0.32; older adults: β_Cognition_=.23, 95% CI 0.11-0.34 and β_Self-efficacy_=.23, 95% CI 0.13-0.34) compared to individuals with lower levels of resource gains who tended to experience higher levels of stress under similar circumstances (ie, younger adults: β_Cognition_=.40, 95% CI 0.21-0.60 and β_Self-efficacy_=.40, 95% CI 0.30-0.50; older adults: β_Cognition_=.37, 95% CI 0.23-0.50 and β_Self-efficacy_=.43, 95% CI 0.30-0.55). While there were no significant results regarding social resources for younger adults, older adults with high levels of social gains experienced less stress when they also presented high levels of social losses (β_Social_=.18, 95% CI 0.04-0.32) compared to older adults with lower levels of resource gains who experienced higher levels of stress under similar circumstances (β_Social_=.45, 95% CI 0.23-0.67).

This study also revealed a significant influence of internet use on the relationship between resource losses and stress levels in both younger and older adults, as indicated by the 2-way interaction effect (internet use × losses). Specifically, the interaction effect was found to be significant for different types of resources in younger and older adults. Among younger adults, a significant interaction effect was observed for self-efficacy and social resources (β_Self−efficacy_=.30, 95% CI –0.02 to 0.31; β_Social_=.67, 95% CI 0.09-1.25). Specifically, younger individuals who reported high losses in social and self-efficacy resources and had higher internet use experienced more stress compared to their counterparts with lower internet use. At the same time, younger adults with low levels of social and self-efficacy losses and higher internet use exhibited lower stress levels than those with lower internet use.

In the case of older adults, a significant interaction effect was observed specifically for cognition resources (β_Cognition_=.08, 95% CI 0.00-0.16). Older individuals who experienced fewer losses in cognition and had higher levels of internet use reported lower levels of stress than individuals with lower internet use. In addition, a significant 2-way interaction effect between internet use and gain was marginally significant (β_Cognition_=–.09, 95% CI –0.21 to .02). Older adults who had high gains in cognition resources and high levels of internet use exhibited lower levels of stress than older adults with lower internet use.

Finally, in older adults, internet use influenced the moderating effect of self-efficacy gains on the relationship between self-efficacy losses and stress and the moderating effect of social gains on the relationship between social losses and stress. This was evidenced by the significant 3-way interactions between internet use, self-efficacy gains, and self-efficacy losses (β_Self-efficacy_=.07, 95% CI 0.01-0.14) but also between internet use, social gains, and social losses (β_Social_=.11, 95% CI 0.03-0.19). Specifically, compared to the second model, the third model presented an increase in the index of adjustment (ie, a change in explained variance) of 0.02 (Δ*R*^2^=0.02; *P*=.03) for the self-efficacy model and .03 (Δ*R*^2^=0.03; *P*=.006) for the social model.

**Figure 3:**
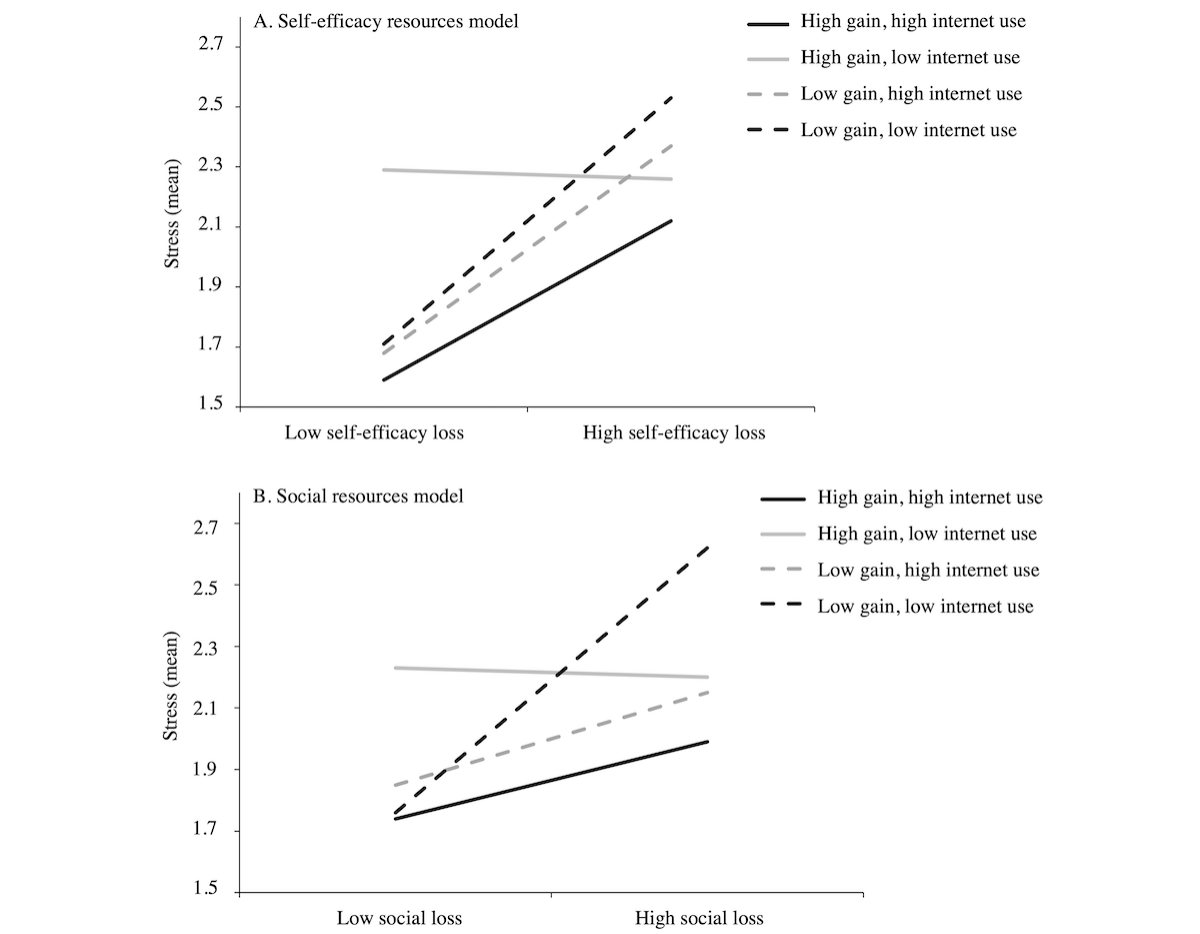
Plots of 3-way interaction effects for the self-efficacy model (self-efficacy losses × self-efficacy gains × internet use) and the social model (social losses × social gains × internet use) in older adults.

It is important to note that in younger adults, regardless of the type of resources, triple interactions between internet use, self-efficacy, cognition or social gains, and self-efficacy, cognition or social losses were not significant, in line with the findings on the triple interaction of the domain-general resources (Multimedia Appendix 4). As the 3-way interaction (losses × gains × internet use) was not significant in younger adults, we reported only the conditional effects of older adults. In older adults, conditional effects analyses (Table 7 and Figure 3) revealed significant moderation effects of self-efficacy gains and internet use on the relationship between self-efficacy losses and stress. Specifically, the moderation effects were observed only when individuals reported high levels of self-efficacy gains and high levels of internet use (β=.29), as well as when individuals reported low levels of self-efficacy gains and high levels of internet use (β=.38) and low levels of both self-efficacy gains and internet use (β=.45). Moreover, significant moderation effects of social gains and internet use on the relationship between social losses and stress were observed only when individuals reported high levels of social gains and high levels of internet use (β=.19) and low levels of both social gains and internet use (β=.64). Regarding domain-general resources, these results suggest that the use of the internet increases the buffering effect of self-efficacy and social gains on the relationship between self-efficacy and social losses and stress.

Regarding the domain-general resources, we conducted an additional analysis to examine the differences between older adults with high internet use and those with low internet use in relation to their gains and losses (Table 8). As the 3-way interaction (losses × gains × internet use) was not significant in younger adults, we reported only the conditional effects of older adults. The results showed that the internet use effects (ie, differences between participants with greater internet use and participants with lower internet use) were observed in older individuals who reported high levels of self-efficacy gains and low levels of self-efficacy losses (β=–.21). Moreover, significant internet use effects were observed in cases in which older adults experienced low levels of social gains and high levels of social losses (β=–.18), as well as in older individuals with low levels of social gains and high levels of social losses (β=–.17).

**Table 7:** Conditional effects of domain-specific resource losses (ie, independent variable) at +1 SD and –1 SD of the mean scores of gains and internet use (ie, moderators) in older adults.

		β coefficient (SE; 95% CI)	*P* value
**Self-efficacy**			
	Losses at –1 SD of gains and –1 SD of internet use	0.45 (0.07; 0.31 to 0.59)	.001
	Losses at –1 SD of gains and +1 SD of internet use	0.38 (0.10; 0.18 to 0.58)	.001
	Losses at +1 SD of gains and –1 SD of internet use	–0.02 (0.12; –0.26 to 0.23)	.88
	Losses at +1 SD of gains and +1 SD of internet use	0.29 (0.07; 0.15 to 0.43)	.001
**Social relations**			
	Losses at –1 SD of gains and –1 SD of internet use	0.64 (0.15; 0.35 to 0.93)	.001
	Losses at –1 SD of gains and +1 SD of internet use	0.23 (0.16; –0.09 to 0.54)	.16
	Losses at +1 SD of gains and –1 SD of internet use	–0.02 (0.14; –0.29 to 0.25)	.88
	Losses at +1 SD of gains and +1 SD of internet use	0.19 (0.08; 0.02 to 0.36)	.02

**Table 8:** Conditional effects of internet use (ie, independent variable) at +1 SD and –1 SD of the mean scores of domain-specific resource gains and losses (ie, moderators) in older adults.

		β coefficient (SE; 95% CI)	*P* value
**Self-efficacy**			
	Internet use at –1 SD of gains and –1 SD of losses	–0.01 (0.05; –0.11 to 0.09)	.82
	Internet use at –1 SD of gains and +1 SD of losses	–0.06 (0.07; –0.19 to 0.07)	.39
	Internet use at +1 SD of gains and –1 SD of losses	–0.25 (0.11; –0.46 to –0.04)	.02
	Internet use at +1 SD of gains and +1 SD of losses	–0.05 (0.06; –0.16 to 0.06)	.39
**Social relations**			
	Internet use at –1 SD of gains and –1 SD of losses	0.03 (0.06; –0.08 to 0.15)	.57
	Internet use at –1 SD of gains and +1 SD of losses	–0.17 (0.09; –0.35 to 0.01)	.07
	Internet use at +1 SD of gains and –1 SD of losses	–0.18 (0.08; –0.34 to –0.01)	.03
	Internet use at +1 SD of gains and +1 SD of losses	–0.08 (0.06; –0.19 to 0.04)	.20

## Discussion

### Principal Findings

This study investigated the influence of internet use on the relationship between personal resources and stress in both younger and older adults. The findings indicated that older adults were less stressed than younger adults. Moreover, resource gains moderated the relationship between resource losses and stress, and this effect was similar in both younger and older individuals. Finally, internet use seems to act as a buffer on the dynamics between social and self-efficacy resource losses and stress, amplifying the positive influence of resource gains in reducing the adverse effects of these losses. In older adults, internet use was beneficial as a means of dealing with losses in social and self-efficacy resources.

### Age-Associated Differences in Stress Levels

In support of our first hypothesis, we found that older individuals reported less stress than younger adults. This finding is consistent with those of extant research that has also documented the stress-buffering effect of age among older adults. This phenomenon has been attributed to several personal factors, including cognitive and emotional processing differences between age groups [64,65], greater use of emotion regulation strategies [66,67], and greater life experience and wisdom that allow for more effective coping with stressors [68,69]. Moreover, older adults may be more skilled at regulating their emotions, which may reduce the impact of stressful events on their psychological well-being.

Specifically, stress-inducing situations are related to an increase in negative emotions, and several studies have shown that older adults tend to experience more positive and less negative emotions [70-74]. Therefore, it can be inferred that older adults may possess a greater capacity to regulate and inhibit negative emotions, leading to a reduced impact of stressful events on their psychological well-being. Older adults may further be more resilient than younger adults due to their accumulated life experience and developed coping mechanisms, including proactive problem-solving strategies, effective emotion regulation, and strong sense of personal control and self-efficacy [75-78]. For example, several studies have reported a coping shift during aging to match the constraints experienced and preserve well-being [79-82]. Older adults, who often face a range of losses associated with aging such as declining health, social network changes (eg, death of partner), and retirement, tend to exhibit a greater preference for accommodation, including emotion- and cognition-focused coping. In contrast, younger adults, who typically have fewer losses, displayed a higher preference for assimilation, including problem-focused coping, and actively sought solutions to alleviate stress, reflecting their developmental stage characterized by a stronger drive for achievement, personal growth, and the ability to confront different types of challenges. The observed shift from assimilation to accommodation [19] across the life span suggests a developmental trajectory in coping strategies, with older adults adapting their coping approaches to address the unique challenges and losses they experience. Overall, older adults may be more resilient to stress than younger adults, and their adaptive coping strategies, social support, and emotion regulation strategies may contribute to their ability to maintain psychological well-being in the face of adversity.

### Resource Gains Moderated the Relationship Between Resource Losses and Stress Levels

We found that resource gains moderated the relationship between resource losses and stress levels, confirming our second hypothesis. In line with the COR theory [20], we found that resource gains buffered the negative impact of resource losses on stress levels. More specifically, COR theory emphasizes the significance of resource gains, which have the potential to assist individuals in restoring their resources and avoiding further depletion. The gain paradox posits that individuals who have experienced losses are more likely to recognize and appreciate resource gains. This phenomenon can be attributed to the increased awareness among individuals of the value and importance of resources as a result of experiencing losses, which in turn serves as a motivation for them to actively seek and increase those resources. Consequently, resource gains are crucial in moderating the negative relationship between resource losses and stress levels as individuals with greater resource gains possess better coping abilities and are more likely to recover from losses [21].

Replicating the COR theory by Hobfoll [20], we also found that resource gains buffered the impact of resource losses in both age groups on stress. That the same buffering effect was found in both age groups could be attributed to some universality of the losses-gains dynamic across life phases. The COR theory posits that individuals across the life span share a fundamental drive to accumulate and protect resources as a means of maintaining well-being and minimizing stress [20]. Therefore, the importance of resource gains in mitigating the negative impact of resource losses on stress levels may hold regardless of age.

### Effect of the Relationship Between Internet Use and Domain-Specific Resources on Stress in Younger and Older Adults

In addition, this study revealed distinct patterns of interaction between effect of internet use as an external technical resource and domain-specific personal resources on stress levels among younger and older adults. Our findings suggest that the relationship between social or self-efficacy losses and stress levels in younger individuals is significantly influenced by their use of the internet. Specifically, younger individuals who reported fewer social or self-efficacy losses experienced lower levels of stress when they used the internet, indicating a buffering effect. On the other hand, those who reported higher social or self-efficacy losses exhibited increased levels of stress when they used the internet, suggesting an exacerbating effect. These results highlight the complex interplay among social or self-efficacy losses, internet use, and stress levels in younger individuals. It appears that the internet may serve as a supportive resource for individuals with fewer social losses, providing them with a means for social connection and support [83]. Furthermore, the internet may serve as a resource for providing access to information, support, and opportunities for skill development, which can bolster self-efficacy beliefs and resilience and promote adaptive coping strategies [84-88]. However, for those experiencing higher social or self-efficacy losses, the internet may exacerbate stress. Indeed, the losses in social resources, such as social contacts, are associated with higher feelings of loneliness [89], which contribute to the development of excessive internet use, commonly referred to as internet addiction [90,91]. This pattern of excessive internet use, driven by the absence of social support and challenges in communication and in emotion identification and regulation, is linked to higher levels of stress [92,93]. This suggests that individuals experiencing significant social losses may increase their use of the internet as a compensatory mechanism to mitigate the impact of these losses, resulting in increased stress levels. Moreover, previous research has documented a generational-situated use of the internet, with younger adults using it for leisure activities whereas older individuals’ preferred use of the internet is to facilitate the realization of daily activities such as medical consultations [94,95].

In addition, this study highlights an interesting pattern regarding the relationship among internet use, cognitive losses, and stress levels in older adults. Specifically, older adults with lower levels of cognitive losses who engaged in internet use experienced lower levels of stress compared to those who did not use the internet, suggesting that internet use may serve as a protective means against stress for older adults with fewer cognitive losses. One possible explanation can be that internet use provides opportunities to be engaged in web-based activities for cognitive stimulation, accessing information, or social interaction [8,96], which may help mitigate the negative effects of cognitive losses on stress levels. Considering that higher frequency of digital device use has been associated with fewer subjective cognitive concerns [97-99] and that cognitive losses have been associated with higher levels of stress in older adults [23,100,101], we found that older adults with less cognitive losses who used the internet more were less stressed compared to older adults who did not use the internet, suggesting a buffer effect of internet use on the relationship between cognitive losses and stress.

However, older adults with high levels of cognitive losses experienced similar levels of stress regardless of their internet use. This suggests that the influence of high cognitive losses on stress may remove any potential benefits derived from internet use. It is possible that older adults with high cognitive losses may have difficulties using the internet effectively due to their subjective cognitive losses, which could be explained by the digital distraction hypothesis [102-104]. According to this hypothesis, increased engagement with technology may have detrimental consequences for cognitive processes, manifesting as executive dysfunction characterized by heightened distractibility, superficial cognitive processing, and difficulties in task organization and completion. In addition, technology reliance may contribute to increased forgetfulness by undermining the natural memory systems used for tasks such as navigation or recalling personal information such as phone numbers [102,104]. The detrimental effects of excessive digital engagement on cognitive functioning may override any potential benefits of internet use for stress reduction in older adults with higher cognitive losses.

### Internet Use Moderated the Relationship Between Resource Losses and Gains and Stress Levels in Older Adults

The final hypothesis of our study, which examined the influence of internet use on the relationship between resources and stress, was confirmed for older adults. More specifically, older adults with fewer losses in self-efficacy and social resources and greater gains in these domains experienced lower levels of stress when they engaged in more internet use. This suggests that the internet can be considered as an “amplifier” of the positive effects of resource gains, particularly in terms of self-efficacy and social resources.

Previous studies have reported that internet use by older adults has been associated with decreased loneliness and depression; better social connectedness, self-esteem, and cognitive functioning [105,106]; and improved self-efficacy, self-control, self-determination, and skill development [107-111]. For example, the study by Karavidas et al [110] examined the association between internet use, self-efficacy resources, and life satisfaction among older adults. The results revealed a positive correlation between internet use and life satisfaction. This relationship was mediated by self-efficacy resources, indicating that increased internet use among older individuals was associated with the development of higher self-efficacy skills, which in turn contributed to an improved overall quality of life. The findings suggest that frequent internet use may serve as a platform for older adults to develop and enhance their self-efficacy, leading to greater life satisfaction. Similarly, Chaumon et al [107] found that older adults with functional loss living in long-term care institutions showed a positive impact of internet use on self-sufficiency, self-efficiency, and psychological empowerment [112].

Moreover, the internet facilitates stronger social connections and easier access to social networks, such as through engaging in web-based conversations with new contacts or actively participating in web-based social events [76,105,107,113]. For example, White et al [105] presented a randomized controlled trial to investigate the psychosocial impact of providing internet training and access to older adults. The study involved a sample of older individuals who were randomly assigned to either an intervention group, which received internet training, or a control group that did not receive any intervention. The results showed significant improvements in several psychosocial factors among the intervention group compared to the control group. Specifically, older adults who received internet training reported increased social support, higher levels of social engagement, reduced feelings of loneliness, and enhanced subjective well-being. These findings suggest that providing older adults with internet training and access can have positive effects on their psychosocial well-being.

In line with previous findings, we found that internet use can support the buffer effects from gains in self-efficacy and social resources when individuals have low levels of losses in each type of resource. Moreover, internet use can also substitute the effects of gains in older adults with low gains and high losses. More specifically, our findings demonstrated that individuals who reported high losses and low gains in social resources experienced lower levels of stress when they engaged in more internet use. This can be attributed to the compensatory role of the internet in filling the gaps caused by the limited gains in social resources. Several studies have provided evidence supporting the notion that the internet can compensate for losses in social resources among individuals [114-118]. Older adults who experience a decline in face-to-face social interactions due to factors such as retirement or physical limitations can benefit from web-based social networking platforms. For example, Khoo and Yang [116] conducted a study that examined the impact of social media use on the perception of social support among middle-aged and older adults. The researchers found that using social media platforms for interactions with broader social networks such as friends was as beneficial as using them to connect with family members in terms of enhancing social support.

### Limitations

This study entails certain limitations that warrant consideration. These pertain both to the representativeness of the recruited sample and to the measures used therein [119]. On the one hand, the results should be interpreted cautiously due to potential sampling error. Indeed, stemming from a nonprobabilistic sampling approach, the characteristics of the participants who voluntarily engaged in the study may also influence the variables of interest [119,120]. For instance, participants’ income could influence their willingness to participate in surveys [121], their resource losses and gains, and their internet use [122]. Consequently, the sampling strategy used (ie, snowball volunteer sampling) likely did not capture older individuals marked by resource losses and sociodemographic characteristics (eg, isolation and low socioeconomic status) that are particularly stressful.

On the other hand, the nature of the measures used may introduce limitations. The results should be interpreted with caution when examining age-related differences in the role of internet use. Reflecting on the issue of causality, it is important to consider how the cross-sectional nature of our study limits our ability to ascertain changes in internet use and its effects over time, especially across different age groups [52]. While we identified associations among changes in personal resources, stress levels, and internet use, these findings are not sufficient to establish a causal link or to delineate the temporal evolution of internet use’s impact on stress and personal resources. This caution extends to interpreting the dynamics of internet use across the life span, where cross-sequential research is essential for distinguishing between the effects of aging and those attributable to cohort-specific experiences or generational differences [94].

Moreover, the decision not to include age group as a factor in an interaction analysis was based on significant differences in internet use between younger and older adults. Preliminary findings indicated uniform high use among younger participants, which could confound nuanced age-related interactions with internet use and stress outcomes. Therefore, analyses were conducted separately for each age group to accurately capture distinct use patterns, especially among the older adults who demonstrated a broader range of internet behaviors. This approach helped avoid the confounding effects of uniform use in younger adults. However, it limited the exploration of broader age-related dynamics, potentially affecting a comprehensive understanding of how age influences the relationship between internet use and stress.

In addition, it is essential to recognize that this study’s focus primarily lay on the frequency of internet use, measured equivalently for both younger and older adults. However, this approach disregards the plausible generational disparities in internet use patterns, as noted in previous research [94,123], which might inadvertently introduce errors in measurement [119]. Notably, the lack of statistical significance concerning the impact of internet use on stress in younger adults, a group extensively engaged with the internet, might potentially be attributed to the distinct “youthful” internet use styles, as proposed by Boullier [94]. Indeed, the presence of measurement errors, such as those arising from questionnaire elaboration, can introduce challenges such as ceiling and floor effects [119,124], impeding the identification of statistically significant differences between groups [125]. Moreover, future studies should develop measures to better capture interindividual differences in internet use in younger individuals to further investigate the multifaceted relationship between internet use and different age groups.

Moreover, the consideration of a singular internet use style in measurement limits the possibility of identifying the styles that may be most beneficial for each age group during resource losses. Indeed, the beneficial role of internet use derives from the meanings attributed to its use and the opportunities it offers to address needs [126-128]. Consequently, it would be of great interest for future studies to focus on identifying favorable internet use styles for stress reduction in a context of personal resource loss across different age groups.

### Conclusions

This study examined the effect of internet use on the relationship between personal resources and stress. The findings revealed a nuanced understanding of how digital engagement can serve as a buffer against stress, particularly among older adults who experienced resource losses and gains. Specifically, older adults who frequently used the internet reported less stress when exposed to high levels of both resource losses and gains compared to their counterparts with lower levels of internet engagement. This underscores the importance of internet use in mitigating stress among older adults, highlighting the potential of digital tools in promoting well-being in older populations.

This study’s novel contribution lies in its empirical support for the beneficial role of internet use among older adults within the framework of the COR theory by Hobfoll [20]. By demonstrating that internet use can moderate the effects of resource losses and gains on stress, this research provides valuable insights for developing targeted interventions aimed at leveraging technology to support well-being. Understanding these dynamics will help researchers, practitioners, and policy makers recognize the role of the internet as a facilitator of resource gains and a compensatory mechanism for social deficits in older adults. Encouraging and supporting older adults in using the internet can promote access to valuable resources and enhance their self-efficacy and social connections, ultimately contributing to improved stress management and, more generally, well-being.

## Appendix

Multimedia Appendix 1 Checklist for Reporting Results of Internet E-Surveys.

Multimedia Appendix 2 Pearson correlations (r) between the main variables.

Multimedia Appendix 3 Unstandardized regression coefficients for domain-general resources in younger and older adults.

Multimedia Appendix 4 Unstandardized regression coefficients for domain-specific resources (self-efficacy, cognition, and social relations) in younger and older adults.

## Abbreviations

COR: Conservation of Resources
