# Supplementary material for: Internet Use as a Moderator of the Relationship Between Personal Resources and Stress in Older Adults: Cross-Sectional Study
Source: JMIR Aging. 2024 Jul 19;7:e52555. doi: 10.2196/52555 (PMC11297370; doi:10.2196/52555)
Supplement: Multimedia Appendix 2 [file aging_v7i1e52555_app2.docx]

| Multimedia Appendix 2. Pearson’s Correlations (*r*) Between Main Variables | | | | | | | | | | | | | | | | | |
| --- | --- | --- | --- | --- | --- | --- | --- | --- | --- | --- | --- | --- | --- | --- | --- | --- | --- |
|  | | |  |  |  |  |  |  | Resource Losses | | | | Resources Gains | | | |  |
| Variables | | |  | Age | Gender | Education | Financial Adequacy | Stress | General | Social | Cognition | Self-Eff. | General | Social | Cognition | Self-Eff. | Internet use |
| Age | | | Total | 1 |  |  |  |  |  |  |  |  |  |  |  |  |  |
|  | | | YA | 1 |  |  |  |  |  |  |  |  |  |  |  |  |  |
|  | | | OA | 1 |  |  |  |  |  |  |  |  |  |  |  |  |  |
| Gender | | | Total | -0.12** | 1 |  |  |  |  |  |  |  |  |  |  |  |  |
|  | | | YA | -0.07 | 1 |  |  |  |  |  |  |  |  |  |  |  |  |
|  | | | OA | 0.05 | 1 |  |  |  |  |  |  |  |  |  |  |  |  |
| Education | | | Total | -0.59*** | 0.07 | 1 |  |  |  |  |  |  |  |  |  |  |  |
|  | | | YA | 0.11 | 0.24*** | 1 |  |  |  |  |  |  |  |  |  |  |  |
|  | | | OA | -0.18** | -0.18** | 1 |  |  |  |  |  |  |  |  |  |  |  |
| Financial Adequacy | | | Total | -0.17*** | 0.16*** | 0.02 | 1 |  |  |  |  |  |  |  |  |  |  |
|  | | | YA | -0.05 | 0.10^+^ | 0.06 | 1 |  |  |  |  |  |  |  |  |  |  |
|  | | | OA | -0.18** | 0.19** | -0.23*** | 1 |  |  |  |  |  |  |  |  |  |  |
| Stress | | | Total | -0.32*** | 0.20*** | 0.08^+^ | 0.26*** | 1 |  |  |  |  |  |  |  |  |  |
|  | | | YA | -0.04 | 0.21*** | -0.04 | 0.29*** | 1 |  |  |  |  |  |  |  |  |  |
|  | | | OA | 0.02 | 0.11^+^ | -0.25*** | 0.12^+^ | 1 |  |  |  |  |  |  |  |  |  |
| **Resources Loss** | | | Total | 0.18*** | -0.04 | -0.24*** | 0.12** | 0.32*** | 1 |  |  |  |  |  |  |  |  |
|  | | | YA | -0.05 | 0.05 | -0.14* | 0.19** | 0.48*** | 1 |  |  |  |  |  |  |  |  |
|  | | | OA | 0.01 | -0.06 | -0.19** | 0.12^+^ | 0.36*** | 1 |  |  |  |  |  |  |  |  |
| Social | | Total | 0.05 | -0.06 | -0.15*** | -0.14*** | 0.20*** | 0.76*** | 1 |  |  |  |  |  |  |  |  |
|  | | YA | -0.06 | -0.05 | -0.19** | 0.14* | 0.26*** | 0.69*** | 1 |  |  |  |  |  |  |  |  |
|  | | OA | -0.10 | -0.05 | -0.10 | 0.16* | 0.21** | 0.83*** | 1 |  |  |  |  |  |  |  |  |
| Cognition | | Total | 0.28*** | -0.02 | -0.29*** | 0.08 | 0.21*** | 0.91*** | 0.45*** | 1 |  |  |  |  |  |  |  |
|  | | YA | -0.04 | 0.08 | -0.11^+^ | 0.17** | 0.34*** | 0.88*** | 0.45*** | 1 |  |  |  |  |  |  |  |
|  | | OA | 0.06 | -0.04 | -0.21** | 0.09 | 0.33*** | 0.94*** | 0.67*** | 1 |  |  |  |  |  |  |  |
| Self-Eff. | | Total | 0.09* | -0.02 | -0.16*** | -0.11* | 0.41*** | 0.89*** | 0.39*** | 0.71*** | 1 |  |  |  |  |  |  |
|  | | YA | -0.02 | 0.06 | -0.08 | 0.15* | 0.53*** | 0.86*** | 0.39*** | 0.64*** | 1 |  |  |  |  |  |  |
|  | | OA | 0.03 | -0.07 | -0.18** | 0.11 | 0.40*** | 0.92*** | 0.65*** | 0.79*** | 1 |  |  |  |  |  |  |
| **Resources Gain** | | | Total | -0.27*** | 0.03 | 0.12** | 0.08 | -0.03 | 0.22*** | 0.28*** | 0.18*** | 0.13** | 1 |  |  |  |  |
|  | | | YA | 0.03 | 0.05 | -0.13* | -0.01 | -0.20*** | 0.11^+^ | 0.14* | 0.14* | 0.01 | 1 |  |  |  |  |
|  | | | OA | -0.24*** | -0.07 | -0.05 | 0.13^+^ | -0.01 | 0.44*** | 0.47*** | 0.40*** | 0.33*** | 1 |  |  |  |  |
| Social | Total | -0.28*** | 0.11* | 0.13** | 0.09* | 0.04 | 0.22*** | 0.09 | 0.19*** | 0.19*** | 0.86*** | 1 |  |  |  |  |  |
|  | YA | 0.01 | 0.15* | -0.07 | 0.01 | -0.08 | 0.19** | 0.09 | 0.21*** | 0.14* | 0.85*** | 1 |  |  |  |  |  |
|  | OA | -0.21** | -0.01 | 0.00 | 0.12^+^ | -0.01 | 0.39*** | 0.37*** | 0.36*** | 0.33*** | 0.87*** | 1 |  |  |  |  |  |
| Cognition | Total | -0.19*** | -0.03 | 0.06 | 0.07 | -0.04 | 0.25*** | 0.33*** | 0.19*** | 0.16*** | 0.95*** | 0.72*** | 1 |  |  |  |  |
|  | YA | 0.03 | -0.02 | -0.17** | -0.01 | -0.17** | 0.15* | 0.21*** | 0.13* | 0.07 | 0.94*** | 0.69*** | 1 |  |  |  |  |
|  | OA | -0.22** | -0.10 | 0.06 | 0.12* | 0.00 | 0.44*** | 0.50*** | 0.38*** | 0.32*** | 0.96*** | 0.74*** | 1 |  |  |  |  |
| Self-Eff. | Total | -0.28*** | 0.01 | 0.14** | 0.07 | -0.04 | 0.11* | 0.22*** | 0.11* | -0.01 | 0.93*** | 0.69*** | 0.84*** | 1 |  |  |  |
|  | YA | 0.04 | 0.02 | -0.09 | -0.02 | -0.28*** | -0.05 | 0.06 | 0.04 | -0.19** | 0.89*** | 0.60*** | 0.77*** | 1 |  |  |  |
|  | OA | -0.24 *** | -0.07 | 0.06 | 0.11^+^ | -0.01 | 0.39*** | 0.44*** | 0.36*** | 0.28*** | 0.96*** | 0.75*** | 0.93*** | 1 |  |  |  |
| Internet use | | | Total | -0.70*** | 0.00 | 0.61*** | 0.05 | 0.13** | -0.18*** | -0.02 | -0.26*** | -0.12** | 0.28*** | 0.26*** | 0.23*** | 0.28*** | 1 |
|  | | | YA | 0.09 | -0.02 | 0.13* | -0.10 | -0.06 | -0.11^+^ | -0.02 | -0.13* | -0.09 | 0.01 | -0.02 | 0.03 | 0.02 | 1 |
|  | | | OA | -0.49*** | -0.15* | 0.46*** | -0.08 | -0.17* | -0.08 | 0.02 | -0.12^+^ | -0.10 | 0.26*** | 0.20** | 0.26*** | 0.25*** | 1 |
| *Notes. ^+^p < .10 ; * p < .05 ; ** p < .01 ; *** p < .001.* | | | | | | | | | | | | | | | | | |
