# Supplementary material for: Internet Use as a Moderator of the Relationship Between Personal Resources and Stress in Older Adults: Cross-Sectional Study
Source: JMIR Aging. 2024 Jul 19;7:e52555. doi: 10.2196/52555 (PMC11297370; doi:10.2196/52555)
Supplement: Multimedia Appendix 3 [file aging_v7i1e52555_app3.docx]

Multimedia Appendix 3– Unstandardized Regression Coefficients for Domain-general Resources in Young and Older Adults.

|  | Young Adults (*N* = 275) | | | | | | Older Adults (*N* = 224) | | | | | |
| --- | --- | --- | --- | --- | --- | --- | --- | --- | --- | --- | --- | --- |
| Independent variables | 1^st^ Model | | 2^nd^ Model | | 3^rd^ Model | | 1^st^ Model | | 2^nd^ Model | | 3^rd^ Model | |
|  | *β* | *p.value* | *β* | *p.value* | *β* | *p.value* | *β* | *p.value* | *β* | *p.value* | *β* | *p.value* |
| Age – cov. | .02 | .16 | .01 | .58 | .01 | .61 | -.01 | .27 | -.01 | .15 | -.01 | .20 |
| Gender – cov. | .31 | < .001 | .30 | < .001 | .31 | < .001 | .12 | .14 | .09 | .27 | .06 | .47 |
| Education Level – cov. | -.03 | .15 | -.04 | .07 | -.04 | .07 | -.04 | .08 | -.04 | .06 | -.04 | .07 |
| Financial Adequacy – cov. | .21 | < .001 | .20 | < .001 | .20 | < .001 | .04 | .68 | .03 | .73 | .02 | .78 |
| Losses | .45 | < .001 | .55 | < .001 | .55 | < .001 | .37 | < .001 | .48 | < .001 | .42 | < .001 |
| Gains | -.20 | < .001 | -.23 | < .001 | -.23 | < .001 | -.13 | .012 | -.12 | .019 | -.06 | .39 |
| Internet Use | .07 | .66 | -.03 | .85 | -.06 |  | -.03 | .50 | -.03 | .47 | -.08 | .10 |
| Losses x Gains | - |  | -.26 | < .001 | -.26 | < .001 | - |  | -.16 | < .001 | -.23 | < .001 |
| Losses x Internet Use | - |  | .41 | .05 | .36 | .15 | - |  | .03 | .50 | .03 | .51 |
| Gains x Internet Use | - |  | - |  | -.02 | .94 | - |  | - |  | -.04 | .37 |
| Losses x Gains x Internet Use | - |  | - |  | .22 | .54 | - |  | - |  | .09 | .05 |
| *R^2^* | .32 | < .001 | .36 | < .001 | .36 | < .001 | .20 | < .001 | .25 | < .001 | .26 | < .001 |
