# Supplementary material for: Internet Use as a Moderator of the Relationship Between Personal Resources and Stress in Older Adults: Cross-Sectional Study
Source: JMIR Aging. 2024 Jul 19;7:e52555. doi: 10.2196/52555 (PMC11297370; doi:10.2196/52555)
Supplement: Multimedia Appendix 4 [file aging_v7i1e52555_app4.docx]

Multimedia Appendix 4– Unstandardized Regression Coefficients for Domain-specific Resources (Self-efficacy, Cognition, and Social Relations) in Young and Older Adults.

|  |  | Young Adults (*n* = 275) | | | | | | Older Adults (*n* = 224) | | | | | |
| --- | --- | --- | --- | --- | --- | --- | --- | --- | --- | --- | --- | --- | --- |
| Independent Variables | | 1st Model | | 2nd Model | | 3rd Model | | 1st Model | | 2nd Model | | 3rd Model | |
|  |  | *β* | *p.value* | *β* | *p.value* | *β* | *p.value* | *β* | *p.value* | *β* | *p.value* | *β* | *p.value* |
| **Social relations** | Age – cov. | .02 | .33 | .01 | .58 | .01 | .57 | -.01 | .46 | -.00 | .53 | -.00 | .64 |
|  | Gender – cov. | .35 | < .001 | .36 | < .001 | .36 | < .001 | .10 | .25 | .07 | .40 | .06 | .49 |
|  | Education Level – cov. | -.03 | .19 | -.05 | .006 | -.05 | .07 | -.05 | .02 | -.05 | .012 | -.05 | .02 |
|  | Financial Adequacy – cov. | .27 | < .001 | .28 | < .001 | .28 | < .001 | .03 | .76 | .03 | .75 | .04 | .64 |
|  | Losses | .21 | < .001 | .23 | < .001 | .22 | < .001 | .21 | .002 | .32 | < .001 | .26 | < .001 |
|  | Gains | -.10 | .012 | -.11 | .006 | -.11 | .008 | -.06 | .24 | -.05 | .26 | -.02 | .71 |
|  | Internet Use | -.05 | .77 | -.10 | .59 | -.09 | .61 | -.05 | .26 | -.05 | .23 | -.05 | .26 |
|  | Losses x Gains | - |  | -.10 | .59 | -.10 | .14 | - |  | -.13 | .009 | -.17 | < .001 |
|  | Losses x Internet Use | - |  | .67 | .02 | .73 | .02 | - |  | -.01 | .89 | -.04 | .53 |
|  | Gains x Internet Use | - |  | - |  | -.22 | .28 | - |  | - |  | -.04 | .33 |
|  | Losses x Gains x Internet Use | - |  | - |  | .12 | .76 | - |  | - |  | .11 | .006 |
|  | *R^2^* | .17 | < .001 | .19 | < .001 | .19 | < .001 | .12 | < .001 | .15 | < .001 | .18 | < .001 |
|  |  |  |  |  |  |  |  |  |  |  |  |  |  |
| **Self-efficacy** | Age – cov. | .02 | .23 | .01 | .54 | .01 | .62 | -.01 | .24 | -.01 | .14 | -.01 | .20 |
|  | Gender – cov. | .29 | < .001 | .29 | < .001 | .29 | < .001 | .14 | .09 | .11 | .18 | .08 | .32 |
|  | Education Level – cov. | -.04 | .13 | -.04 | .07 | -.04 | .05 | -.04 | .09 | -.04 | .06 | -.04 | .07 |
|  | Financial Adequacy – cov. | .23 | < .001 | .23 | < .001 | .23 | < .001 | .03 | .76 | .02 | .81 | .01 | .87 |
|  | Losses | .32 | < .001 | .30 | < .001 | .30 | < .001 | .31 | < .001 | .35 | < .001 | .29 | < .001 |
|  | Gains | -.14 | < .001 | -.15 | < .001 | -.15 | < .001 | -.07 | .09 | -.06 | .13 | -.00 | .98 |
|  | Internet Use | .06 | .71 | -.04 | .71 | -.03 | .85 | -.03 | .38 | -.04 | .30 | -.09 | .05 |
|  | Losses x Gains | - |  | -.08 | .015 | -.08 | .02 | - |  | -.10 | .005 | -.15 | < .001 |
|  | Losses x Internet Use | - |  | .30 | .06 | .33 | .04 | - |  | .01 | .69 | .04 | .34 |
|  | Gains x Internet Use | - |  | - |  | .06 | .72 | - |  | - |  | -.06 | .12 |
|  | Losses x Gains x Internet Use | - |  | - |  | .15 | .36 | - |  | - |  | .07 |  |
|  | *R^2^* | .36 | < .001 | .38 | < .001 | .38 | < .001 | .23 | < .001 | .25 | < .001 | .26 | < .001 |
|  |  |  |  |  |  |  |  |  |  |  |  |  |  |
|  |  | Young Adults (*n* = 275) | | | | | | Older Adults (*n* = 224) | | | | | |
|  |  | 1st Model | | 2nd Model | | 3rd Model | | 1st Model | | 2nd Model | | 3rd Model | |
|  |  | *β* | *p.value* | *β* | *p.value* | *β* | *p.value* | *β* | *p.value* | *β* | *p.value* | *β* | *p.value* |
| **Cognition** | Age – cov. | .02 | .27 | .02 | .37 | .02 | .37 | -.01 | .25 | -.01 | .03 | -.01 | .19 |
|  | Gender – cov. | .28 | < .001 | .28 | < .001 | .28 | < .001 | .10 | .21 | .10 | .23 | .07 | .39 |
|  | Education Level – cov. | -.05 | .07 | -.05 | .06 | -.05 | .06 | -.04 | .09 | -.04 | .10 | -.03 | .11 |
|  | Financial Adequacy – cov. | .25 | < .001 | .24 | < .001 | .24 | < .001 | .06 | .53 | .07 | .41 | .05 | .52 |
|  | Losses | .26 | < .001 | .31 | < .001 | .31 | < .001 | .28 | < .001 | .34 | < .001 | .28 | < .001 |
|  | Gains | -.15 | < .001 | -.16 | < .001 | -.16 | < .001 | -.09 | .08 | -.06 | .50 | .05 | .56 |
|  | Internet Use | .07 | .69 | .03 | .88 | -.00 | .98 | -.03 | .43 | -.03 | .40 | -.08 | .09 |
|  | Losses x Gains | - |  | -.09 | .08 | -.10 | .08 | - |  | -.08 | .04 | -.20 | .002 |
|  | Losses x Internet Use | - |  | .14 | .46 | .10 | .63 | - |  | - |  | .12 | .01 |
|  | Gains x Internet Use | - |  | - |  | -.04 | .86 | - |  | - |  | -.09 | .09 |
|  | Losses x Gains x Internet Use | - |  | - |  | .15 | .51 | - |  | - |  | .08 | .12 |
|  | *R^2^* | .22 | < .001 | .23 | < .001 | .23 | < .001 | .18 | < .001 | .21 | < .001 | .22 | < .001 |
